# Mixture Design and Doehlert Matrix for the Optimization of the Extraction of Phenolic Compounds from *Spondias mombin* L Apple Bagasse Agroindustrial Residues

**DOI:** 10.3389/fchem.2017.00116

**Published:** 2018-01-05

**Authors:** Antonio C. Santos Felix, Cleber G. Novaes, Maísla Pires Rocha, George E. Barreto, Baraquizio B. do Nascimento, Lisandro D. Giraldez Alvarez

**Affiliations:** ^1^Grupo de Pesquisa Aromas e Análise de Alimentos, Departamento de Ciências e Tecnologias, Universidade Estadual do Sudoeste da Bahia, Jequié, Brazil; ^2^Grupo de Pesquisa Laboratório de Química Analítica, Departamento de Ciências e Tecnologias, Universidade Estadual do Sudoeste da Bahia, Jequié, Brazil; ^3^Departamento de Nutrición y Bioquímica, Facultad de Ciencias, Pontificia Universidad Javeriana, Bogotá, Colombia; ^4^Instituto de Ciencias Biomédicas, Universidad Autónoma de Chile, Santiago, Chile

**Keywords:** *Spondias mombin* L., Cajá, polyphenols, factorial design, ABTS, DPPH

## Abstract

In this study, we have determined, using RSM (mixture design and Doehlert matrix), the optimum values of the independent variables to achieve the maximum response for the extraction of total phenolic compounds from *Spondias mombin* L bagasse agroindustrial residues in order to preserve their antioxidant activity. The extraction of phenolic compounds, as well as their antioxidant capacity and the capacity to scavenge ABTS, was determined by the modified DPPH method at different periods of time, temperature, velocity of rotation and solvents concentration. We observed that the optimum condition for the highest antioxidant yield was obtained using water (60.84%), acetone (30.31%), and ethanol (8.85%) at 30°C during 20 min at 50 rpm. We have also found that the maximum yield of total phenolics was 355.63 ± 9.77 (mg GAE/100 g), showing an EC_50_ of 3,962.24 ± 41.20 (g fruit/g of DPPH) and 8.36 ± 0.30 (μM trolox/g fruit), which were measured using DPPH and ABTS assays. These results suggest that RSM was successfully applied for optimizing the extraction of phenolics compounds thus preserving their antioxidant activity.

## Introduction

The consumption of fruit pulps has been highly recommended since dietary phytochemicals, such as phenolic compounds and carotenoids. Also have been associated with the prevention of degenerative and chronic diseases such as cancer, neurodegenerative diseases, metabolic syndrome-related disorders, and inflammation (Tanaka et al., 2012; Del Rio et al., 2013). Recently, increased attention has been given to the fruits industry residues as abundantly available and cheap renewable feedstock for the production of value-added compounds like polyphenols (Sójka et al., 2013; Zhu et al., 2015). In Brazil, fresh fruits are consumed during the year or commercialized as frozen pulp. For example, *Spondias mombin* L. (Cajá) is distributed throughout Brazil, where this exotic fruit is known by a wide variety of regional names like cajá, taperebá, and cajá miúdo. Cajá belongs to the Anacardiaceae family and is found in the tropical areas of Asia, Africa, and America. This fruit is rich in vitamins, and C, calcium, phosphorus, tannins, potassium, and carotenoids and can be consumed in natura or in the form of pulp, nectar, sorbet, jam, or liqueur (Lima et al., 2011; Tiburski et al., 2011). It can be found in this fruit high levels of carotenoids and phenolic compounds, providing cajá high functional and nutritional value that may help to prevent various diseases, including cardiovascular disorders (Wang et al., 2011). Previous studies showed that (all-*E*)-zeinoxanthin, (all-*E*)-lutein and (all-*E*)-β-cryptoxanthin are the main carotenoids in the saponified extract of cajá pulp (Hamano and Mercadante, 2001; Tiburski et al., 2011).

Phenolic compounds are plant secondary metabolites commonly found in plants and derived products such as citrus fruit, berries, legume seeds, cocoa, apples, grapes, olives, tomatoes, onions, broccoli, soybeans, lettuce, grains and cereals, white and red wines, green and black teas, and coffee beans (Birt et al., 2001; Alu'datt et al., 2017; Magalhães et al., 2017). It is difficult to develop an ideal method for extraction of all phenolic compounds, since the polarity may vary significantly. Thus, the optimization of variables is essential to obtain better extraction of phenolic compounds from different food matrices (Garcia-Salas et al., 2010). Variables such as extraction cycles, time and temperature, solvent type, solvent acidity, solvent concentration used in the process can influence the efficiency of extraction of total phenolic compounds and the antioxidant capacity (Mokrani and Madani, 2016; Tomšik et al., 2016). The combination of solvents with different polarities has been recommended for an efficient extraction of phytochemicals (Rufino et al., 2010; Martins et al., 2013). For example, Pulsed Electric Field pretreatment can significantly enhance the extraction process (Quagliariello et al., 2016). In addition, cavitation phenomena and mechanical mixing effect using ultrasound-assisted extraction increase extraction efficiency and reduces extraction time (Ince et al., 2012).

Generally, there is no extract or compound that can be used as universal antioxidant. Therefore, it is necessary the search for new sources and specific antioxidants for such purpose. In this sense, the optimization of the extraction of phenolic compounds is essential to reach an accurate analysis, and this process has been traditionally performed using the one-variable-at-a-time method (OVATM). However, this technique does not allow assessing the effects of interactions between variables (Bezerra et al., 2016). In the last years, multivariate chemometric tools such as Response Surface Methodology (RSM) are useful for optimizing specific compound extraction (Novaes et al., 2017). In addition, the application of RSM provides a faster and less expensive procedure to obtain optimal values of the variables affecting the extraction (De Souza et al., 2014). Some studies on the optimization of the extraction of bioactive phenolic compounds from fruits using RSM have been reported (Celli et al., 2015; Saikia et al., 2015; Simić et al., 2016; Gomes et al., 2017). This study has as aim to estimate the effects of several process variables, including solvent concentration, stirring speed, time and temperature for the extraction of total phenolic compounds using RSM (mixture design and Doehlert matrix) and antioxidant activity from *Spondias mombin* L. (Cajá) bagasse agro-industrial residues.

## Materials and methods

### Chemicals

DPPH• (2,2-diphenyl-1-picrylhydrazyl radical), ABTS, 2,2 azino bis (3-ethylbenzo thiazoline 6 sulfonic acid), gallic acid monohydrate (98%), sodium carbonate P.A, Folin-Ciocalteu's phenol reagent and Trolox (2,5,7,8-tetramethylchroman-2-carboxylic acid), were purchased from Sigma-Aldrich Chemical Company (São Paulo, SP, Brazil). Acetone P.A., distilled water and 95% ethanol P.A. were obtained from Vetec (Rio de Janeiro, RJ, Brazil). 99.8% Methanol P.A. was purchased from Chemis (São Paulo, SP, Brazil).

### Sample

Cajá apple bagasse agro-industrial residues were collected in May 2015 in local industry (Frutisol, Jequié, Bahia, Brazil, geographical coordinates: 13°52′13′ South latitude, 40°9′31′ West longitude). Residues were dried in an air-circulating oven for 72 h at 50°C, and then the dried residues were ground with a knife mill (model SL31, SOLAB). The powder obtained was sieved through a 20-mesh size screen and stored at 20°C until extraction and analysis.

### Extraction of phenolic compounds

Cajá apple bagasse agro-industrial residues (10 g), 5-fold volume (w/v) were pitted and homogenized with 50 mL of the mixture (ethanol, acetone, and water) with a shaking incubator (Shaker SL 222, Solab). A mixture design was performed to optimize the best proportion of the three solvents used. Afterwards, Doehlert matrix was used to optimize the variables time (10, 30, 60, 90, and 110 min), temperature (10, 20, 30, 40, and 50°C) and rotation speed (50, 150 e 250 rpm) that significantly affect the extraction process. The factors optimized by mixture design remained constant during execution of the Doehlert design. All determinations, performed randomly, were carried out in triplicate and the data recorded as mean ± SD. The experimental domain, expressed as coded and real values for each factor, and the response (Total Phenolic) obtained are shown in Table 1. Designs and experimental data were processed using the statistic software 10® (The StatSoft, Inc., Tulsa, OK, USA) with 95% of confidence level. After completion of the extraction time, the crude extract was centrifuged at 5,000 rpm (Fanem-Tecnal, São Paulo, Brazil) for 10 min. The extract was filtered on paper for removal of solids particles, and the supernatant was collected and analyzed for apparent phenolic content, ABTS, and DPPH• radical scavenging activity.

**Table 1:** Design matrix and results for optimization of the extraction of compounds phenolic.

**Run**	**Variables**			Total Phenolic^*^(mg GAE/100 g)	**Total Phenolic Predicted**
	**Acetone (%)**	**Water (%)**	**Ethanol (%)**		
**MIXTURE DESIGN**					
1	100.0 (1)	0.0 (0)	0.0 (0)	119.81 ± 3.80	120.41
2	0.0 (0)	100.0 (1)	0.0 (0)	155.77 ± 4.90	153.27
3	0.0 (0)	0.0 (0)	100.0 (1)	124.41 ± 6.90	125.40
4	50.0 (½)	50.0 (½)	0.0 (0)	170.32 ± 9.30	170.77
5	50.0 (½)	0.0 (0)	50.0 (½)	124.60 ± 4.20	128.57
6	0.0 (0)	50.0 (½)	50.0 (½)	168.35 ± 10.4	169.22
7	66.7 (2/3)	16.7 (1/6)	16.7 (1/6)	150.90 ± 10.3	147.63
8	16.7 (1/6)	66.7 (2/3)	16.7 (1/6)	166.10 ± 14.70	172.13
9	16.7 (1/6)	16.7 (1/6)	66.7 (2/3)	153.33 ± 2.00	148.79
10 (CP)	33.3 (1/3)	33.3 (1/3)	33.3 (1/3)	171.26 ± 13.6	163.91
11(CP)	33.3 (1/3)	33.3 (1/3)	33.3 (1/3)	155.96 ± 19.89	163.91
12 (CP)	33.3 (1/3)	33.3 (1/3)	33.3 (1/3)	167.14 ± 14.50	163.91
**Run**	**Variables**			Total Phenolic^*^(mg GAE/100 g)	**Total Phenolic Predicted**
	**Time (min)**	**Temperature (°C)**	**Agitation speed (rpm)**		
**DOEHLERT DESIGN**					
1	0 (30.0)	+1.68 (110.0)	0 (150)	111.37 ± 15.45	138.67
2	−1 (20.0)	+1 (90.0)	−1 (50)	173.25 ± 24.17	174.33
3	− (20.0)	+1 (90.0)	+1 (250)	130, 02 ± 44.78	144.31
4	+1 (40.0)	+1 (90.0)	−1 (50)	230.73 ± 51.52	281.00
5	+1 (40.0)	+1 (90.0)	+1 (250)	294.14 ± 45.28	183.20
6	−1.68 (10.0)	0 (60.0)	0 (150)	164.77 ± 6.43	150.91
7 (CP)	0 (30.0)	0 (60.0)	0 (150)	229.56 ± 29.07	261.36
8 (CP)	0 (30.0)	0 (60.0)	0 (150)	268.80 ± 37.90	261.36
9 (CP)	0 (30.0)	0 (60.0)	0 (150)	286.17 ± 76.16	261.36
10	(1.68) 50.0	0 (60.0)	0 (150)	215.13 ± 38.58	228.71
11	−1 (20.0)	−1 (30.0)	−1 (50)	321.61 ± 48.17	269.73
12	−1 (20.0)	−1 (30.0)	+1 (250)	107.47 ± 53.43	171.94
13	+1 (40.0)	−1 (30.0)	−1 (50)	307.03 ± 53.08	308.63
14	+1 (40.0)	−1 (30.0)	+1 (250)	178.68 ± 41.73	210.84
15	0 (30.0)	−1.68 (10.0)	0 (150)	212.42 ± 47.78	184.72

CP, Central Point;

* *Mean ± Standard Deviation*.

### Total phenolic determination

Total phenolics content was quantified according to the adapted Folin–Ciocalteu method (Rebaya et al., 2015). Extracts (0.5 mL) were mixed with 2.5 mL of Folin–Ciocalteu reagent (1:10) and 2 mL of 4% (w/v) sodium carbonate solution. The mixture was stirred and kept at room temperature for 2 h in the dark. The absorbance of the solution of each sample was measured at 750 nm using a UV–Vis spectrophotometer (Marte Spectro 560). A blank solution containing all reagents without the sample or the gallic acid at the same conditions was also measured. Gallic acid in aqueous medium was used for calibration. Results were expressed as milligrams of gallic acid equivalents per 100 g of residue (mg GAE/100 g). All measurements were performed in triplicate.

### Determination of antioxidant activity

Different methods have been developed to assay free radical scavenging capacity and total antioxidant activity. Generally, these methods involve the determination of the disappearance of free radicals using spectrophotometry technique. In this work, two methods (DPPH• scavenging activity for estimation the free radical scavenging properties and ABTS^+^) were used to assess the total antioxidant capacity.

#### Scavenging ability toward DPPH radical

DPPH method was used in the determination of the antioxidant capacity, which is based on the quantification of free radical-scavenging with modifications. This method depends on the reduction of DPPH• radical (purple) to a yellow colored diphenyl picrylhydrazine. A decrease in the DPPH absorbance indicates an increase of the DPPH• radical scavenging activity (Abdel-Hameed, 2009). A methanolic solution containing 0.06 mM of the DPPH• radical was prepared daily and protected from light. 0.1 mL of fruit extract was added to 3.9 mL of DPPH• methanolic solution. The decrease in absorbance at 515 nm using a UV–Vis spectrophotometer was measured at 1 min intervals for the first 10 min, and then at 5 min intervals until stabilization. All measurements were performed in triplicate. The antioxidant capacity was expressed as EC_50_ index, defined as the amount of antioxidant needed to decrease the initial DPPH• radical concentration by 50% and values expressed as g fruit/g DPPH•.

#### ABTS^+^ assay

The ABTS^+^ assay was performed according to method established previously with modifications (Re et al., 1999). The pre-formed radical monocation (ABTS^+^•) was produced by oxidation of 7 mM ABTS stock solution with 145 mM potassium persulfate and then incubated in the dark for 16 h at room temperature before use. The ABTS^+^ working solution was prepared by diluting the stock solution with ethanol until reach an absorbance of 0.70 ± 0.02 (at 734 nm). All samples were diluted approximately to provide 20–80% inhibition of the blank absorbance. 30 μL of the extract was mixed with 3.0 mL ABTS^+^ working solution. The absorbance of the mixture was measured at 734 nm after 6 min of incubation at room temperature. The ABTS scavenging capacity was expressed as μM trolox/g fruit.

## Results and discussion

The two largest groups of phenolic compounds are phenolic acids and flavonoids. These molecules can modulate the expression and activity of several enzymes in cell signaling and metabolism (Tan et al., 2011). The antioxidant effect can be attributed to the reducing power of aromatic hydroxyl groups, which reduce the activity of highly reactive species and free radicals (Pereira et al., 2012). Thus, exploring and understanding the phytochemical composition of native fruits are desirable for the search of new functional food sources.

### Optimization of the extraction process

In this study, we have determined the optimum values of the independent variables to achieve the maximum response for the extraction of total phenolics compounds from *Spondias mombin* L bagasse agroindustrial residues, thus preserving its antioxidant activity. Two response surface methodology techniques were used: (1) mixture design and (2) Doehlert matrix. The extraction of phenolic compounds, as well as their antioxidant capacity and capacity to scavenge ABTS, was determined by the modified DPPH method at different periods of time, temperature, velocity of rotation and solvents concentration.

#### Mixture design

In order to optimize the extraction process, a mixture design was developed as shown in Table 1. When working with mixtures variables, this freedom of combination between levels does not exist, because they cannot vary without taking into consideration the levels of other variables. The variables, in this case, represent the components of a mixture and the sum must always be constant and equal to 100% (Novaes et al., 2017). The design was performed to optimize the best proportion of the three solvents used (ethanol, acetone, and water). In this regard, the variables time and temperature were fixed in 2 h and 25°C, respectively.

The recovery of phenolic compounds depends on the polarity of the solvent used; therefore, in this work, we used ethanol, water, and acetone with a dipolar moment (μ_r_) of 1.69 Debye (D), 1.85 D and 2.88 D, respectively (Martins et al., 2013). Karami et al. (2014) used response surface methodology (RSM) to optimize extraction condition of phenolic compounds from licorice root by microwave application and Soxhlet extraction. The authors proved that microwave–assisted extraction is a more effective technique compared to the conventional method (soxhlet), but this technique is very time consuming and requires relatively large quantities of solvents. In our experimental model, extraction could be completed in few hours with high reproducibility, thus reducing the consumption of solvent and energy due to its shorter time (Karami et al., 2014).

**Figure 1:**
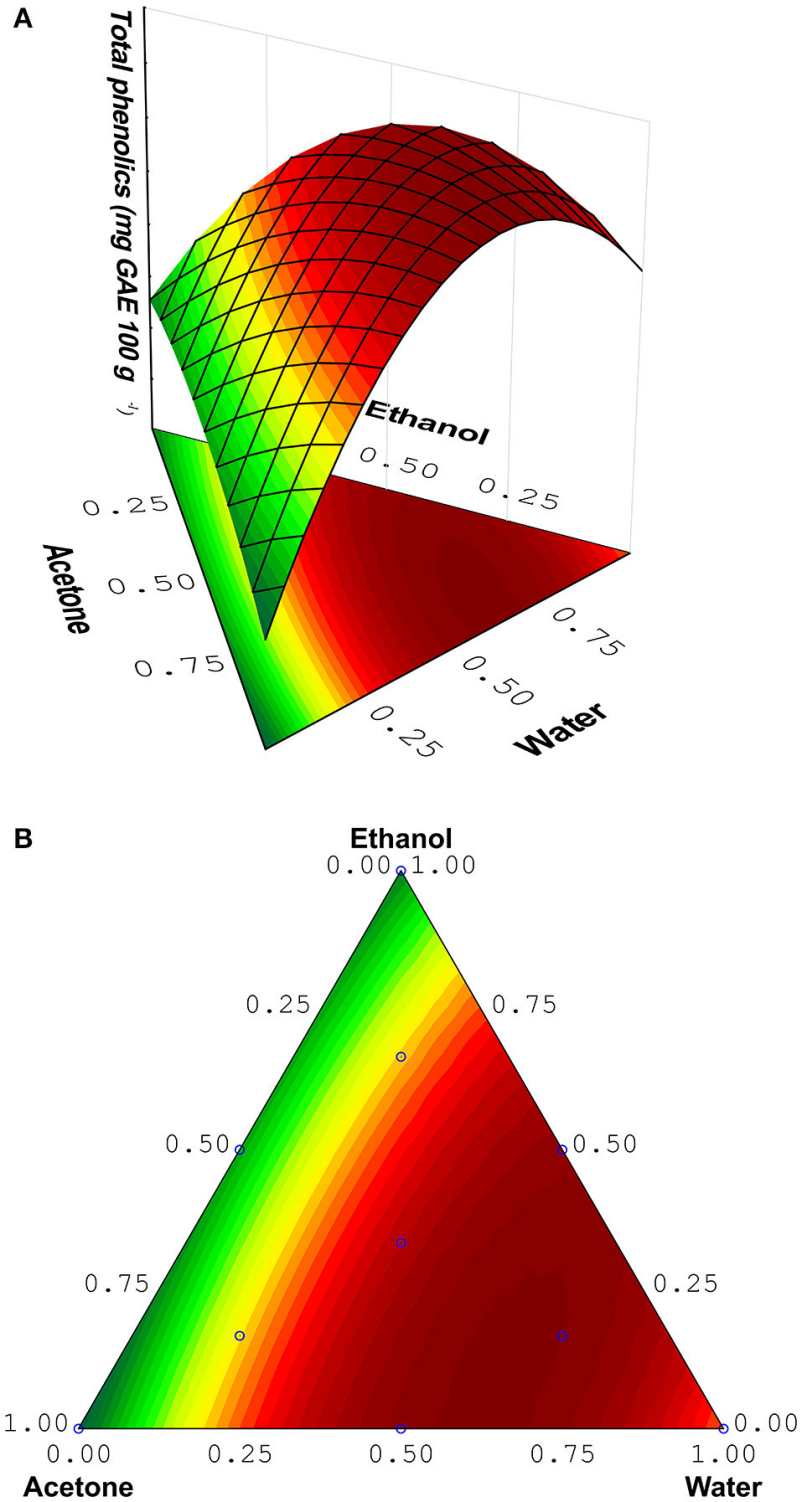
**(A)** Response surface and **(B)** Contour plot obtained from Mixture design.

The response surface obtained by mixture design is illustrated in Figure 1A, and contour plot is illustrated in Figure 1B. This surface presented a maximum as critical point. The coordinates of this point are the proportions of the solvents that generate the greatest response. The proportions optimized according to the response surface were water (60.8%), acetone (30.3%), ethanol (8.9%), being the maximum inside the experimental domain. These values correspond to water (30 mL), acetone (15 mL) and ethanol (5 mL) approximately, for a final volume of 50 mL. The lowest contents of phenolic compounds were obtained using 100% acetone (run 1), 100% ethanol (run 3) or mixture 50% acetone/50% ethanol (run 5), while the highest were obtained with water only or mixture of water and acetone/ethanol. This occurs due to the wide range of phenols that the aqueous mixtures can dissolve and increased solvation provided by water. The combined use of water and organic solvent may facilitate the extraction of molecules that are soluble in water and/or organic solvent. For example, Turkmen et al. (2007) found that aqueous solvents were more efficient in extracting total phenolics from black tea (Turkmen et al., 2007). According to Alothman et al. (2009) acetone (50%) was the most efficient solvent for extracting phenols from honey pineapple, while acetone (90%) efficiently extracted phenols from the Thai seedless guava (Alothman et al., 2009). Indeed, Zhou et al. (2017) developed an ultrasound-assisted extraction (UAE) method to extract natural antioxidants from Melastoma sanguineum Sims, and RSM was used to optimize the conditions of UAE to maximize the extraction efficiency. In this study, the authors demonstrated that UAE was a more efficient method for extracting natural antioxidants from this fruit in comparison to conventional extraction methods (Soxhlet), (Zhou et al., 2017).

The coordinates of the maximum point are found using the first derivative of the mathematical function (Lemos et al., 2009). The better proportion of the solvents to the extraction of phenolics can also be observed by surface shown in Figure 1. The equation correlating the three variables and the analytical response is:

$$\begin{array}{l} \text{TP}{=}^{*}120.41(\text{A}){+}^{*}153.27(\text{W}){+}^{*}125.43(\text{E}){+}^{*}135.72(\text{AW})\\ \;+22.61(\text{AE}){+}^{*}119.49(\text{WE}) \end{array}$$

The values marked with ^*^ are considered significant; TP, Total phenolics; A, Acetone; W, Water; and E, Ethanol. Design matrix and the response obtained are shown in Table 1 (*n* = 3).

Statistical analysis was carried out with the experimental values and the main effects of the variables. The analysis of the main effects and their interactions in the form of analysis of variance (ANOVA) are presented in Table 2 at the 95% confidence level (*p* < 0.05). The *p*-values are used to check the significance of the corresponding coefficient and the smaller the *p*-values are, the bigger the significance of the corresponding coefficient (De Lima Da Silva et al., 2009). The model presented a *p*-value 0.001, which indicates that the model is very suitable. The Lack-of-Fit was insignificant, due to high *p*-value (0.8153) and lower *F*-value (0.38). The non-significant lack-of-fit corroborated the good predictability of the model. All the three principal factors studied, and their interactions were significant except for the AE interaction (Acetone × Ethanol), which had a value (0.4176) more than 0.05. In addition, the analysis of the residues generated between the predicted values and the observed values, the coefficient determination value (0.9448) and adjust coefficient of determination value (0.8988) indicated a good predictability of the model. Li et al. (2017) established a microwave-assisted extraction (MAE) method to extract antioxidants from the fruit of Gordonia axillaris, and the method was compared with two conventional methods (Soxhlet extraction and maceration extraction). The antioxidant capacity of the extract determined by MAE was stronger than those obtained by Soxhlet extraction (114.1 ± 2.0 μmol Trolox/g DW) or maceration (168.7 ± 3.9 μmol Trolox/g DW) (Li et al., 2017).

**Table 2:** ANOVA for the models.

**Sources of variations**	**Sum of squares**	**Degree of freedom**	**Mean square**	***F*-value**	***P*-value**
**MIXTURE DESIGN**					
^*^Model	3, 761.82	5	752.36	20.53	0.001
Total Error	219.84	6	36.64		
Lack of Fit	94.49	4	23.62	0.37	0.815
Pure Error	125.35	2	62.67		
Total Adjusted	3, 981.66	11	361.97		
**DOEHLERT DESIGN**					
Temperature (Te)	3, 865.99	1	3, 865.99	4.43	0.128
^*^(Te)^2^	11, 907.78	1	11, 907.78	13.65	0.034
Time (Ti)	7, 425.28	1	7, 425.28	8.51	0.061
(Ti)^2^	6, 145.00	1	6, 145.00	7.04	0.076
Velocity of rotation (VR)	6, 458.15	1	6, 458.15	7.40	0.072
(VR)^2^	295.13	1	295.13	0.33	0.601
(Te)(Ti)	2, 416.53	1	2, 416.53	2.77	0.194
^*^(Te)(VR)	14, 428.74	1	14, 428.74	16.54	0.026
(Ti)(VR)	1, 793.43	1	1, 793.43	2.05	0.247
Lack of Fit	3, 296.11	2	1, 648.05	1.88	0.294
Pure Error	2, 616.48	3	872.16		
Total Adjusted	69, 630.28	14			

* *Significant*.

The application of the mixture design was effective to establish the best proportion among the solvents for the extraction of phenolic compounds, thus contributing to reduce the waste generated in accordance with the principles of the green chemistry (Sheldon, 2012). In addition, conventional extraction methods, such as maceration and Soxhlet extraction presents low efficiency and potential environmental hazards due to the high demand for organic solvents (Brglez Mojzer et al., 2016).

#### Doehlert design

After the preliminary evaluation, a Doehlert design was used to screen three variables (time, temperature and velocity of rotation) that may affect polyphenol extraction. The conditions optimized in the first design were maintained constant: water (60.84%), acetone (30.31%) and ethanol (8.85%). The number of experiments required is given by the expression N = k^2^ + k + C_o_, where k is the number of variables and *C*_*o*_ is the number of center points (Ferreira et al., 2004). This design is represented by a geometric solid, characterized by alternating square faces with triangular faces and allows assessing different numbers of levels for each studied variable. Table 1 showed the matrix of the experimental design of the three factors expressed as coded and real values and the response (Total Phenolic - TP) obtained (*n* = 3). The factors time (Ti), temperature (Te) and velocity of rotation (VR) were varied from 10 to 50 min, 10.0 to 110.0°C and 50 to 250 rpm, respectively. The function that represented the relationship between the factors (Ti, Te, and VR) and Total Phenolic (TP) for polyphenol extraction from cajá is as follows:

$$\begin{array}{l} \text{TP}=357.83-0.51(\text{Te}){-}^{*}0.04{\left(\text{Te}\right)}^{2}+6.22(\text{Ti})-0.18{\left(\text{Ti}\right)}^{2}\\ \;{-}^{*}2.21(\text{VR})+0.0010{\left(\text{VR}\right)}^{2}+0.07(\text{Te})(\text{Ti})\\ \;{+}^{*}0.017(\text{Te})(\text{VR})+0.018(\text{Ti})(\text{VR}) \end{array}$$

**Figure 2:**
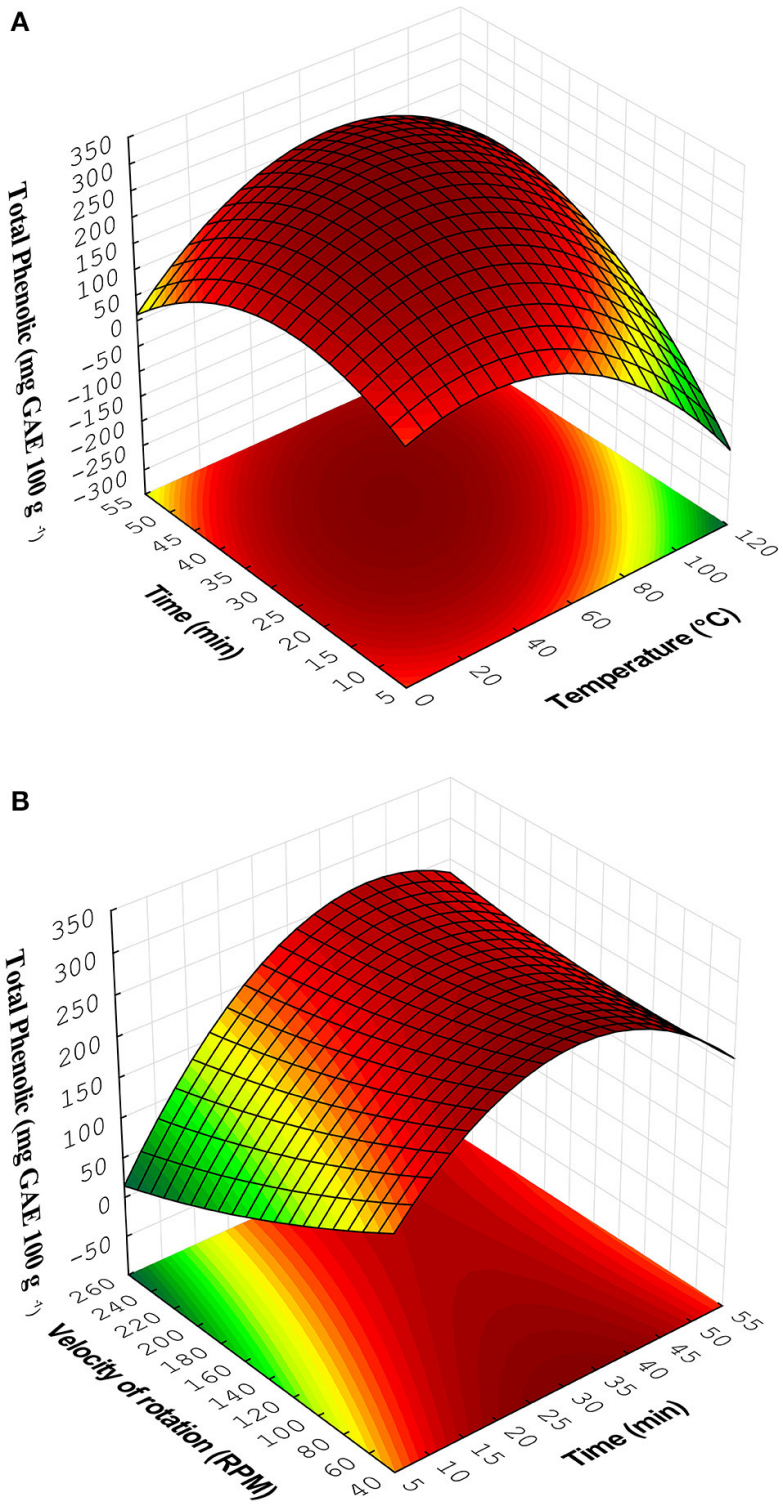
Response surfaces obtained from Doehlert matrix for **(A)** Time × Temperature and **(B)** Velocity of rotation × Time.

The values marked with ^*^ are considered significant. This model fitted the experimental data. Figure 2A showed the effect of time extraction and temperature and Figure 2B illustrated the effect of time extraction and velocity of rotation in the extraction of phenolic compounds. The critical points were calculated by solving the equation system formed by the partial derivatives of the function (Martendal et al., 2007). The critical points can also be observed by visual inspection of the charts depicted in Figures 2A,B.

$$\begin{array}{l} \;\partial \text{TP}/\partial (\text{Te})=\text{0=-0}.\text{51-0}.\text{08}(\text{Te})-0.\text{07}(\text{Ti})+0.\text{017}(\text{VR})\\ \;\partial \text{TP}/\partial (\text{Ti})=0=6.22+0.07(\text{Te})-0.36(\text{Ti})+0.018(\text{VR})\\ \partial \text{TP}/\partial (\text{VR})=0=\text{ ​}-2.21\text{​}+\text{​}0.017(\text{Te})\text{​}+\text{​}0.018(\text{Ti})\text{​}+\text{​}0.0020(\text{VR})\\ \;(\text{Te})=67^{{}^{\circ}}\text{C},(\text{Ti})=40\text{min and }(\text{VR})\text{=}180\text{rpm} \end{array}$$

Figure 2A showed an increase in total phenolic content with the increase of extraction time at fixed time, while Figure 2B demonstrated an increase in total phenolic content with the lowest velocity of rotation. Best extraction of phenols was obtained at intermediate level of temperature. The extraction time had this effect because increasing the contact time of the solvent with solids may improve the diffusion of the compounds (Corrales et al., 2009) and high temperatures improve the efficiency of the extraction; however, the excessive temperature may degrade phenolic compounds (Piñeiro et al., 2016). The highest contents of total phenolic were observed in experimental run 11 (Ti = 20 min, Te = 30°C and VR = 50 rpm), while the lowest yield of total phenolic compounds was observed in the run 12 (Ti = 20 min, Te = 30°C, and VR = 250 rpm).

ANOVA was carried out with the experimental values and the main effects of the variables at the 95% confidence level (Table 2). The lack of fit was not significant at the confidence level of 95%. This finding was checked from the *F*-test, where the ratio MS (mean of square)_lack of fit_/MS (mean of square)_pure error_ for the quadratic equation was equal to 1.89, a lower value than the tabled *F*_(3, 2)_ at the confidence level of 95% [*F*_(3, 2)_ = 19.16] (Gorla et al., 2016).

### Effects of conditions on antioxidant activity

We found that the maximum yield of total phenolic was 355.63 ± 9.77 (mg GAE/100 g), showing an EC_50_ of 3,962.24 ± 41.20 (g fruit/g of DPPH) and 8.36 ± 0.30 (μM trolox/g fruit), which were measured by DPPH and ABTS assays, respectively. The antioxidant capacity of the extracts was obtained under optimal conditions: water (60.84%), acetone (30.31%), and ethanol (8.85%) at 67°C during 40 min at 180 rpm. For instance, ABTS assay estimates more accurately the antioxidant capacity of foods, particularly those containing hydrophilic, lipophilic and highly pigmented compounds (Floegel et al., 2011). Meanwhile, the DPPH assay is employed to test the ability of compounds to act as free radical scavengers and frequently used to evaluate the antioxidant capacity of foods. When a solution of DPPH• radical is mixed with an antioxidant substance, its color turns from purple to yellow (Pyrzynska and Pekal, 2013). One of the ways to improve the extraction of antioxidants is increasing the solvent temperature. However, one of the major problems regarding the antioxidant capacity of the extracts is to preserve their stability. In spite of that, the results from this study demonstrated that cajá bagasse agroindustrial residues can be considered as a potential source of phenolic bioactive compounds with potential antioxidant capacity. In the literature, there are not many data for cajá, and the values for antioxidant activity present differences between the methods, and this caveat did not allowed us a proper comparison. However, regarding our optimal extraction conditions, we were able to obtain an important antioxidant capacity in comparison to other fruits. For example, De Souza et al. (2014) found an EC_50_ of 3,778.94 ± 333.88 (g fruit/g of DPPH) and 7.87 ± 0.87 TEAC (μM/g fruit) when the extracts of strawberry were obtained with methanol/water (50:50, v/v) at room temperature for 1 h. Furthermore, Rufino et al. (2010) observed an EC_50_ of 9,397 ± 64.8 (g fruit/g of DPPH) and 7.8 ± 0.2 TEAC (μM/g fruit) and C50 of 1,064 ± 162 (g fruit/g of DPPH) and 40.7 ± 2.2 TEAC (μM/g fruit) in aqueous-organic extracts, based on fresh or dry matter, respectively. In this present study, we have found parameters that can obtain not only the maximum extraction of total phenolic, but also an effective antioxidant capacity by using a short period of time (20 min). It should be noteworthy that antioxidants can exert protective roles against free radicals by a variety of mechanism including catalytic systems to neutralize or scavenge reactive oxygen species (ROS) (Hwang et al., 2015). It is worthwhile to highlight that we used bagasse residues. The recovery of antioxidants from these residues are interesting from a technological point of view as valuable components of nutraceuticals in food and pharmaceutical preparations or in the cosmetics industry. In addition, can also be useful to attenuate the environment damage and better exploited the food production chain (Van Der Goot et al., 2016). Natural products present an attractive source of chemical structures with promising pharmacological profiles. In this concern, Ajaegbu et al. (2016) determined the adulticidal activity of *S.mombin* leaf extract and fractions against female of *Aedes aegypti* mosquitoes suggesting that *Spondias mombin* leaf extract and fractions may be utilized for the development of plant-based pesticides as an alternative to synthetic insecticides (Ajaegbu et al., 2016).

## Conclusion

The importance of this work is that we were able to obtain the optimal conditions for the maximum extraction yield of polyphenolic compounds with potential antioxidant capacity. We determined that the optimum condition for the highest antioxidant yield was obtained using water (60.8%)/acetone (30.3%)/ethanol (8.9%) mixture at 67°C during 40 min at 180 rpm. This method was also easier and cheaper than other methods to perform polyphenols extractions since does not require expensive reagents or high quantities of organic solvents. Fruit sources like waste cajá may provide new natural products into the food industry with safer and better antioxidants qualities against oxidative damage.

## Author contributions

All authors listed, have made substantial, direct and intellectual contribution to the work: AS and MP have made all the experiments. CN and GB have made all the statistical procedures. BdN and LG have made discussion.

### Conflict of interest statement

The authors declare that the research was conducted in the absence of any commercial or financial relationships that could be construed as a potential conflict of interest.
